# Anorexia and Bulimia Nervosa in Spanish Middle-Aged Adults: Links to Sociodemographic Factors, Diet, and Lifestyle

**DOI:** 10.3390/nu16162671

**Published:** 2024-08-13

**Authors:** Elena Sandri, Eva Cantín Larumbe, Germán Cerdá Olmedo, Gaia Luciani, Stefano Mancin, Marco Sguanci, Michela Piredda

**Affiliations:** 1 Faculty of Medicine and Health Sciences, Catholic University of Valencia San Vicente Mártir, c/Quevedo, 2, 46001 Valencia, Spain; elena.sandri@ucv.es (E.S.); german.cerda@ucv.es (G.C.O.); 2 Doctoral School, Catholic University of Valencia San Vicente Mártir, c/Quevedo 2, 46001 Valencia, Spain; 3 Faculty of Data Science, Polytechnical University of Valencia, Camí de Vera s/n, 46022 Valencia, Spain; evacantinlarumbe@gmail.com; 4 Department of Biomedicine and Prevention, Tor Vergata University, Via Montpellier, 1, 00133 Rome, Italy; gaia.luciani.gl@gmail.com; 5 IRCCS Humanitas Research Hospital, Via Manzoni 56, 20089 Milan, Italy; stefano.mancin@humanitas.it; 6 Department of Biomedical Sciences, Humanitas University, Via Rita Levi Montalcini 4, 20090 Milan, Italy; 7 Research Unit of Nursing Science, Department of Medicine and Surgery, Campus Bio-Medico di Roma University, Via Alvaro del Portillo, 21, 00128 Rome, Italy; marco.sguanci@unicampus.it

**Keywords:** feeding and eating disorders, feeding behavior, healthy lifestyle, survey, Spain

## Abstract

Aim: This study examines the prevalence of eating disorders, particularly anorexia and bulimia nervosa, among middle-aged Spaniards, noting their rising incidence in men as well as women. It explores how these disorders relate to sociodemographic factors and lifestyle habits. Methods: A cross-sectional survey was conducted from August 2020 to November 2021 via online platforms. Participants were Spanish nationals aged 31–50 years residing in Spain. Results: Out of 9913 respondents, 96.66% reported no diagnosed eating disorders, while 3.34% reported one or more (0.36% anorexia nervosa; 0.53% bulimia nervosa; 1.97% other eating disorders; 0.48% multiple disorders). Concerns about body image and lack of control over food intake were reported by 50% and 28%, respectively, suggesting potential undiagnosed disorders. Significant BMI differences were noted between healthy individuals and those with anorexia or bulimia. Conclusions: Anorexia and bulimia affect both adolescents and middle-aged individuals, challenging existing stereotypes. The high prevalence of undiagnosed eating disorder behaviors highlights the need for early detection. To address these issues in Spain, targeted awareness programs are essential to reduce stigma and improve service access through clinical, social, and political collaboration.

## 1. Introduction

Eating Disorders (EDs) present an intricate spectrum of psychiatric conditions with far-reaching implications for mental and physical well-being. Classified within the comprehensive category of “feeding and eating disorders” in the Diagnostic and Statistical Manual of Mental Disorders (DSM-5-TR), these conditions arise from dysfunctional eating behaviors disrupting not only the consumption or absorption of food but also leading to compromised physical health and psychosocial functioning [1]. The DSM-5-TR categorizes EDs into six distinct groups, prominently featuring Anorexia Nervosa, Bulimia Nervosa, and Binge-Eating Disorder, and providing a framework to comprehend the diverse presentations and manifestations of these disorders [1].

The expanding body of literature consistently reinforces the link between EDs and a decrease in quality of life, heightened disease risks, susceptibility to depression, substance abuse, and an increased likelihood of suicidal tendencies [2]. Individuals navigating EDs often encounter a distinctive set of challenges, marked by ambivalence towards treatment, limited illness awareness, and an underestimation of symptom severity [3].

Clinically, individuals with EDs present with a spectrum of physiological consequences, including dehydration, intestinal ptosis, thinning and hypotonia of the visceral muscle layer due to malnutrition, alterations in hypothalamic temperature regulation, loss of subcutaneous adipose tissue, thinning of the epidermis-dermis layer, and disruptions in the hypothalamic-pituitary-gonadal axis due to protein-energy malnutrition and insufficient essential body fat [4,5]. Frequent elimination behaviors, such as self-induced vomiting and misuse of laxatives, diuretics, and enemas, contribute to hydroelectrolytic imbalances, significantly increasing the risk of hypokalemia and associated cardiac and renal complications [5].

Within the field of eating disorders, two of the most important and prevalent are anorexia nervosa and bulimia [2]. The former is characterized by extreme weight loss due to self-imposed starvation and an intense fear of gaining weight, often accompanied by a distorted body image [1]. Symptoms include severely restricted eating, excessive exercise, and, in some cases, binge eating and purging [5]. The second involves cycles of binge eating followed by compensatory behaviors such as self-induced vomiting, excessive exercise, or laxative misuse. Individuals with bulimia usually maintain a normal weight but engage in these behaviors at least once a week for three months [1].

Traditionally, the literature emphasized a higher prevalence of EDs among females, linked with Western cultural influences. However, the last decade, characterized by cultural globalization, has witnessed a shift, with numerous studies showing an increasing prevalence of these disorders also among males and in Eastern countries, challenging established stereotypes and emphasizing the need for a nuanced understanding [6]. Accordingly, the comprehensive review by Galmiche et al. [7] revealed prevalence rates of 4.6% in North America, 3.5% in Asia, and 2.2% in Europe.

Predisposing conditions range from familial influences and early-life adversities to societal pressures and emotional states, emphasizing the importance of recognizing and comprehending these factors in devising effective preventive and therapeutic strategies [8]. Studies in this domain draw attention to the influential role of traditional family structures in shaping attitudes and behaviors related to eating [9]. The narrative extends to encompass maternal criticisms as substantial influencers in the ED landscape. These critiques, directed towards body shape, weight, and eating habits, emerge as potent catalysts, fostering body dissatisfaction and contributing to disordered eating patterns across all genders [6]. The correlation between EDs and specific sporting activities adds another layer of complexity. Weight-category sports (martial arts, wrestling, boxing), endurance sports (long-distance running, cycling, swimming, cross-country skiing), and those emphasizing individual performance (dance, figure skating, artistic gymnastics) exhibit heightened associations with ED development [10].

Eating disorders, once predominantly associated with younger populations, have garnered increasing attention in the context of middle-aged adults, suggesting a growing prevalence both in women and men [11,12,13]. Unique considerations for individuals navigating eating disorders in their middle years are also identified [14] have explored the notion of perimenopausal eating disorders, emphasizing the intersection of physiological changes during midlife and disordered eating behaviors. Henriksen et al. [15] studied the clinical presentation of adult women seeking specialty eating disorder treatment, investigating whether age influences the manifestation of ED symptoms. Body image perceptions, along with the intricate interplay of societal, familial, and peer influences, may contribute to eating disorders among middle-aged women [16,17]. Although increasing attention to EDs in middle-aged adults, the literature is still sparse. In particular, no epidemiological study was found on the prevalence and related factors of EDs in the middle-aged Spanish population. Therefore, this study aimed to describe the prevalence of eating disorders and their relationship with sociodemographic variables, nutrition habits, and lifestyle habits of the Spanish middle-aged population.

## 2. Materials and Methods

### 2.1. Study Design and Sampling

A cross-sectional study was conducted with the Spanish middle-aged population. The sample included individuals aged between 31 and 50 years of Spanish nationality and resident in Spain. Subjects with chronic illnesses that could potentially impact their dietary habits and those who were currently experiencing situations that temporarily disrupted their usual diet, such as hospitalization or prison admission, were excluded. Potential participants were approached via email or social media and encouraged to share the link to the online survey with their contacts through the “snowball” method [18]. This involves starting with participants who meet the study’s criteria and then asking them to identify others who might also qualify and be interested. This process continues until enough participants are reached. The study adheres to the STROBE Guidelines [19].

### 2.2. Ethical Considerations

The study adhered to the principles outlined in the Declaration of Helsinki and was approved by the Research Ethics Committee of the Catholic University of Valencia San Vicente Martir (approval code UCV/2019-2020/152, 18 June 2020). Potential participants were provided information about the study aim and procedure and asked for their consent to study participation and data handling. They were informed that the data would be collected anonymously and that the data would be analyzed in an aggregated way. The participants provided their informed consent to study participation and data handling before filling out the questionnaire.

### 2.3. Instrument and Variables

The self-report questionnaire used in the study included three sections: 1. nutrition habits (frequency of consumption of various groups of food); 2. health-related social habits (including exercise, smoking, alcohol consumption, sleep patterns); 3. self-reported anthropometric (weight, height) and sociodemographic data (gender, age, place of residence, occupation, education, income level). Moreover, self-perceived health status and symptoms of eating disorders were collected.

The instrument was previously developed and psychometrically tested [20] in accordance with a rigorous methodology [21]. Content validity was evaluated involving an expert panel (*n* = 7) including a nutritionist, two family physicians, two psychologists, a social educator, and a communication expert. Face validity was ascertained through a pilot study with 53 subjects with characteristics similar to those of the study population.

The majority of the study’s variables were qualitative, offering respondents a range of options from which to choose. These included factors like the frequency of food and drink consumption, sedentary lifestyle habits, hours of sleep, tobacco use, gender (male or female), place of birth and residence, and educational attainment (ranging from basic education, including no education, primary or secondary education, vocational training or baccalaureate; higher education including bachelor’s, master’s or doctoral degrees), with income categorized as either low or medium–high based on household income thresholds (<2200 EUR per month for low income; >2200 EUR per month for medium–high income). In addition to qualitative variables, there were quantitative continuous variables such as age, weight, height (reported by participants), and the number of minutes spent in sports activities per week. Furthermore, certain variables were discretely quantitative, measured using a 5-point Likert scale, such as self-reported health status. The variables concerning the frequency of food consumption (including fruit, vegetables, meat, dairy, cereals, legumes, and soft drinks) were employed to calculate a condensed and validated version of the IASE (healthy-eating index for the Spanish population) [22]. This index assesses how regularly individuals consume foods recommended for daily and weekly intake, as well as those intended for occasional consumption. Moreover, it recognizes dietary diversity as a crucial aspect of maintaining a healthy diet. Behaviors in alignment with the recommendations of the Spanish Society of Community Nutrition (SENC) are assigned a score of 10 [23]. The maximum score achievable on the IASE is 73. Based on the IASE score, the nutritional habits of the population can be classified into three groups: scores falling between 58.4 and 73 are categorized as “Healthy”, scores between 36.5 and 58.4 are classified under “Needs changes”, and scores below 36.5 are labeled as “Unhealthy”. Variables related to nutrition and health habits that were not covered by the IASE were classified, as in the case of previous articles [24,25], using a 4-point Likert scale (ranging from 1 for no or low frequency to 4 for maximum frequency), except for body mass index (BMI) and minutes of exercise, which were treated as numerical values. This classification approach offers a systematic evaluation of health-related behaviors, enabling a detailed comprehension of participants’ habits within these domains. Finally, the variables pertaining to potential symptoms of eating disorders encompassed worries about feeling or becoming fat (which we called “Obesophobia”), difficulty controlling food intake or feeling ashamed after eating (“No control”), and concerns about body shape (“Body image”). These frequency-related variables were organized using a 6-point Likert scale (ranging from 6 for “always” to 1 for “never”). Additionally, the survey directly asked participants if they had received a diagnosis of any eating disorders.

### 2.4. Data Collection

The instrument was built as an online form and disseminated through email contacts and personal networks in different social media. The main social channel was Instagram, where the account @elretonutricional was created for this study. Several nutrition and health professionals, influencers, and supporters were involved to help with dissemination. Moreover, emails were sent to several establishments across Spain, such as pharmacies and tobacconists, selected because of their diverse clientele. Data were collected from August 2020 to November 2021.

### 2.5. Statistical Analyses

The questionnaire responses were compiled into a dedicated database and carefully scrutinized to address any errors or inconsistencies, particularly focusing on issues related to data entry and outliers. Furthermore, certain variables underwent categorization or calculation based on other variables. Extreme BMI values (below 14 and above 40) were removed to ensure data robustness. Discrete variables were presented as absolute values and percentages (prevalence), while continuous variables were summarized using mean and standard deviation. Subsequently, descriptive and inferential statistical analyses were conducted. To assess the normality of the data, the Lilliefors Test (Kolmogorov–Smirnov) was employed at a 95% significance level, indicating a departure from normal distribution. Therefore, non-parametric statistical tests (Mann–Whitney, Kruskal–Wallis) were used to analyze continuous variables, and the χ^2^ test was used for categorical variables. A Dunn test with a post-hoc Bonferroni correction was applied to provide further insights into the differences identified among multiple groups or pairs. A *p*-value < 0.05 was considered statistically significant. All statistical analyses and plots were carried out using Python 3.9.

## 3. Results

The study involved a final sample of 9913 individuals of Spanish nationality and residing in Spain, aged between 31 and 50 years. Most participants were female (83.36%), on average 39.35 years old, highly educated (72.73%), with a medium–high income level (54.78%), and lived in a city with more than 10.000 inhabitants (79.35%). More details of the sample sociodemographic characteristics are outlined in Table 1.

Among the participants, 96.66% (*n* = 9582) reported not having diagnosed eating disorders (ED), while 3.34% (*n* = 331) stated that they had one or more diagnosed eating disorders. Specifically, 0.36% (*n* = 36) reported a diagnosis of anorexia nervosa (AN), 0.53% (*n* = 53) of bulimia nervosa (BN), 0.48% (*n* = 48) reported more than one ED, finally the remaining 1.97% (*n* = 194) reported other types of ED. Table 2 presents the percentage of each response relative to the entire population for each diagnosed eating disorder.

Results regarding eating disorder symptoms for the whole sample are presented in Table 3. It is striking that 50.4% of the respondents claimed to be frequently, very frequently, or always worried about body image. Similarly, 49.5% of the respondents claimed to be afraid of becoming fat (Obesophobia) frequently or very frequently (24.3% “frequently” + 14.3% “very frequently” + 10.9% “always”). A similar trend, although with lower prevalence, was observed for having “No control” over food intake, reported frequently by 28.1% of the sample.

The prevalence of respondents affected by anorexia or bulimia who reported at least frequently possible symptoms of eating disorders was even higher than those of the entire sample, as expected (Table 4). In particular, they reported worrying about body image (79.8%), obesophobia (80.8%), and lack of control over food intake (58.4%).

Separate analyses were conducted for the health, social habits, and lifestyle variables, namely body mass index (BMI), healthy-eating index, frequency of consumption of various food groups, smoking, sleep quality, and propensity for physical activity between three distinct groups of respondents, differentiated based on the diagnosed eating disorder (1. Healthy population; 2. Population affected by anorexia nervosa; 3. Population affected by bulimia nervosa) (Table 5).

Significant differences in BMI were found between the healthy population and the individuals diagnosed with anorexia nervosa (AN) or bulimia nervosa (BN); however, no significant differences were observed between individuals with either of the two eating disorders. Regarding the healthy-eating index (IASE), no statistically significant differences were identified among the groups.

Regarding the frequency of consumption of fried and fast food, statistically significant differences were observed only between the healthy population and those with anorexia nervosa, while for the consumption of ultra-processed foods and fish, no statistically significant differences were found between the groups. Examining the relationships between the three studied groups and the frequency of beverage consumption, no significant differences emerged regarding the consumption of water, sugary drinks, fruit juices, and coffee or energy drinks. No statistically significant differences were found among the three groups, neither regarding health status nor sedentary lifestyle. However, individuals with anorexia nervosa engaged in significantly more physical exercise compared to healthy individuals and individuals with bulimia nervosa. Regarding sleep habits, restful awakening was reported without significant differences among the groups. However, individuals with bulimia nervosa reported shorter sleep duration compared to healthy individuals. Interestingly, despite the shorter sleep duration of individuals with bulimia nervosa, there were no statistically significant differences in sleep quality between the groups. Examining variables related to social habits, no differences were observed between the groups regarding alcohol consumption, smoking, or nighttime activities. However, it emerged that individuals with BN appear to be more prone to episodes of getting drunk compared to healthy individuals.

Regarding sociodemographic variables (Table 6), both anorexia nervosa and bulimia nervosa were significantly more common among women than men, with most of AN cases (*n* = 35) and BN cases (*n* = 51) identified among female participants. No statistically significant differences were found among the groups for other sociodemographic variables considered, such as education level, income level, and size of the city of residence.

**Figure 1:**
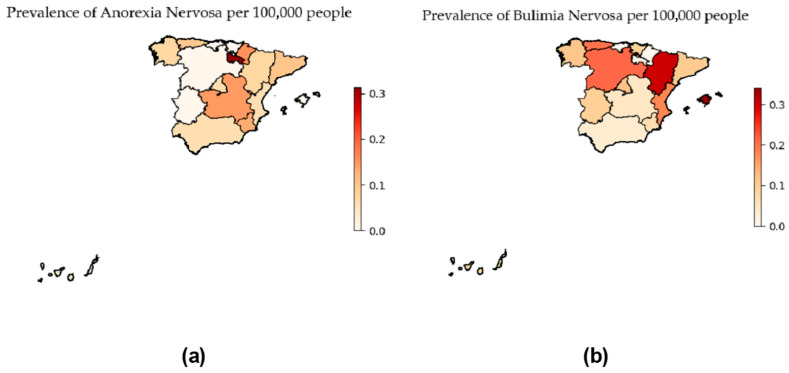
Prevalence of eating disorders based on regions in Spain: (**a**) Anorexia prevalence per 100,000 people; (**b**) Bulimia prevalence per 100,000 people.

Finally, Figure 1 shows the prevalence of Anorexia nervosa and Bulimia nervosa in the different regions of Spain. No significant differences were found between the Autonomous Communities neither for AN (*p*-value = 0.64, Kruskal–Wallis), nor for BN (*p*-value = 0.45, Kruskal–Wallis).

## 4. Discussion

The study aimed to examine the association between diet, lifestyle habits, and sociodemographic factors with diagnosed eating disorders and possible eating disorder symptoms in the middle-aged Spanish population. The prevalence of diagnosed eating disorders among participants was 3.34% for all disorders, 0.36% for anorexia nervosa (AN), and 0.53% for bulimia nervosa (BN). These disorders were primarily observed in female gender, as most participants were female. However, a small male minority was also identified. These results are consistent with previous research [2], which highlighted a higher prevalence of eating disorders among women due to Western cultural influences. However, in recent years, there has been an increase in the prevalence of these disorders among men [26,27], which makes it even more interesting to study their sociodemographic and behavioral determinants. Comparison of the prevalence of “Obesophobia”, “No control”, and “Body image” between subjects with diagnosed eating disorders and the entire sample might suggest the presence of individuals in limbo between predisposition and actual development of an eating disorder or who may already suffer from one ED without having received a formal diagnosis. Almost half of the entire sample, including the great majority (96.6%) of individuals who consider themselves “healthy” (i.e., not diagnosed with EDs), exhibit at least frequent concerns about weight gain. These results reflect the complexity of eating disorders and suggest the presence of undiagnosed subpopulations that may require particular attention [28,29]. The prevalence of these concerns may be influenced by contemporary sociocultural changes, such as the idealization of thinness promoted by the media and by constant body comparison on digital platforms, factors that can contribute to the spread of eating disorders, and the challenge of early diagnosis [30,31]. Frequent concern about weight gain or experience of shame after eating can be signs of an eating disorder, particularly Anorexia Nervosa [32], and may be associated with a distorted perception of body image or dysfunctional perfectionism [33]. Such personality traits can lead to a persistent pursuit of thinness ideals, constant measurement, and self-demanding, thus contributing to the manifestation and maintenance of eating disorders [34,35]. These behaviors can be particularly dangerous when combined with low self-esteem, increasing the risk of developing eating disorders [34,36,37,38]. As for Bulimia Nervosa, similar behaviors may be observed, although with some differences in manifestation mode. For example, individuals with BN often experience binge-eating episodes followed by compensatory behaviors such as self-induced vomiting, laxative use, or excessive exercise [39]. These behaviors can be triggered by the same distorted perception of body image and dysfunctional perfectionism that characterize AN. Similarly, these behaviors may be associated in BN with an obsessive search for weight and body shape control, which in turn can fuel the cycle of eating disorders [40]. As with obesophobia, more than half of the entire sample reported at least frequent concern about body image. Body dissatisfaction is commonly identified as a significant risk factor for EDs. Individuals who are dissatisfied with their bodies tend to be more prone to develop disordered eating behaviors such as binge eating and purging to achieve greater satisfaction and conform to social thinness ideals [41]. Eating disorders have complex origins, influenced by psychological factors, social pressures, and cultural expectations often linked to body image and self-esteem [42]. The relationship between city size and eating disorders is intricate and depends on a series of interconnected factors [43,44,45]. Data from this study suggest that living in a large city compared to a small city may not significantly influence the prevalence of eating disorders. This is supported by previous research [2], which highlighted that the demographic diversity present in large cities may mitigate the effect of city size on the prevalence of eating disorders. Moreover, small cities can offer robust health resources and social networks that reduce disparities in mental health, including eating disorders [46]. Furthermore, access to healthcare services does not seem to be strictly correlated with city size. Small cities may have highly accessible and well-integrated healthcare services within the community, which can be equally effective in preventing and treating eating disorders compared to large cities [46]. Social pressures related to body image and cultural expectations may be present in both large and small cities, leading to a similar prevalence of eating disorders in both [42]. Although urbanization can have negative consequences on mental health, the likelihood of living in a metropolis being a primary risk factor for developing an eating disorder appears improbable [42]. However, when combined with other sociodemographic factors, it is plausible that the risk of developing an eating disorder may escalate [43]. In particular, the study by van Son and colleagues [44] concluded that urban life is a potential environmental risk factor for bulimia nervosa but not for anorexia nervosa. In conclusion, the relationship between city size and eating disorders is confirmed as complex. While there may be some differences, current data indicate that city size may not be the decisive factor in the prevalence of eating disorders. Further research is needed to fully understand this complex relationship. There were no statistically significant differences in the prevalence of anorexia nervosa or bulimia nervosa in relation to the participants’ level of education or income. This homogeneity could be explained by several factors. First, the shared food culture in Spain, with its emphasis on the Mediterranean diet and communal dining experiences, may foster a positive and balanced relationship with food among individuals of all socioeconomic backgrounds. This cultural perspective encourages the consumption of fresh, whole foods and promotes social interactions around meals, which can contribute to a healthier approach to eating and potentially reduce the likelihood of developing eating disorders [47]. Second, the increased awareness and accessibility of health and nutrition information, facilitated by the diffusion of the Internet, social media, and various media outlets, allows individuals from diverse socioeconomic groups to learn about healthy-eating practices and the risks associated with eating disorders. This broad dissemination of information empowers individuals to make informed choices about their diet and lifestyle, potentially mitigating the prevalence of eating disorders across different demographic groups [48,49]. Finally, a greater societal understanding and acceptance of eating disorders may lead to improved early detection and intervention efforts, regardless of socioeconomic status. As public discourse surrounding mental health and well-being continues to evolve, individuals from all walks of life may feel more comfortable seeking support and treatment for eating disorders, therefore reducing disparities in access to care and improving overall outcomes [50,51]. Data regarding health factors for the three population groups (healthy individuals, individuals diagnosed with anorexia nervosa, and individuals diagnosed with bulimia nervosa) indicate that the BMI varies significantly between groups. Healthy individuals show a higher BMI compared to individuals with AN, reflecting the extreme food restriction and significant weight loss associated with anorexia [52]. Unlike AN, individuals with BN tend to maintain a weight within the normal range despite weight fluctuations due to binge-eating episodes and compensatory behaviors [1]. However, it is important to note that such weight fluctuations and compensatory behaviors can have serious effects on the physical and mental health of individuals with BN [53]. Regarding eating habits, no significant differences emerged between groups in terms of the healthy-eating index. The scores attained by all three groups fall within a range indicating the necessity for changes. However, there are differences in the consumption of certain types of food. Compared with healthy individuals, those diagnosed with AN tend to consume fewer fried and fast foods than others, as they may perceive them as dangerous for their weight control, and they may experience profound feelings of guilt or shame following the consumption of foods deemed unhealthy, such as fried foods. The avoidance of such foods serves as a protective mechanism aimed at circumventing these adverse emotions [54].

### Strengths and Limitations

This study has notable strengths such as the large sample size that can provide a fairly accurate representation of health behaviors in the Spanish middle-aged population, good geographical representativeness, and diversity of variables analyzed that allows different nutritional, sporting, and lifestyle habits to be related to sociodemographic determinants, offering a more complete picture of the health status of the population screened. Some limitations need to be taken into account. The data were collected through an online survey, which may introduce response bias that was observed in terms of the gender of respondents with eating disorders, the majority of whom were female. It has been observed in multiple studies [55,56,57,58] that women are more inclined to participate in survey-based research, especially in the areas of nutrition and health. This may be attributed to a higher level of concern and sensitivity towards these issues among women compared to men. Bias was also detected in the educational level of the sample. A higher level of education may be associated with a greater concern for health and fitness, which could influence the prevalence of eating disorders. To address these limitations, we are currently conducting a second dissemination of the survey aimed at collecting a larger sample that includes more male respondents and people with a lower level of education. This will provide a more balanced and representative picture of the study population.

## 5. Conclusions

The issues related to EDs, such as AN and BN, are not confined to adolescents but also affect segments of the Spanish population aged between 31 and 50 years. The detailed analysis conducted in this study found that:

1. Concerns about body image and lack of control over food intake were reported by 50% and 28%, respectively, suggesting potential undiagnosed disorders.
2. Significant BMI differences were noted between healthy individuals and those with anorexia or bulimia.
3. A growing prevalence of eating disorders is found among both women and men and across various age groups, challenging entrenched stereotypes.

This underscores the need for a broader and more inclusive view in the research and management of such disorders, especially considering their impact on the overall well-being of the population. While the rate of diagnosed EDs is relatively low, the high prevalence of self-reported behaviors possibly indicates the presence of eating disorders even in the absence of a formal diagnosis, emphasizing the importance of a thorough understanding of such conditions. The urgency of further research on eating disorders lies in their health hazard and long-term sequelae, and the possibility of an underestimated prevalence. The aim is to detect these disorders early to provide timely and effective medical care, thus improving the quality of life of patients and minimizing both the physical and psychological repercussions on affected individuals and their close environment. Furthermore, it is important to emphasize a multidisciplinary and targeted approach in addressing EDs, considering the various factors that may contribute to their onset and maintenance. This includes not only clinical diagnosis and treatment but also prevention and promotion of healthy and mindful lifestyles. To effectively address eating disorders in the age group between 31 and 50 years, it is crucial to adopt an integrated approach that encompasses not only thorough research and analysis of specific risk factors for this population but also the promotion of greater awareness and education regarding mental and dietary health. Moreover, investing in targeted awareness programs and educational initiatives can help reduce the stigma associated with eating disorders and increase accessibility to diagnosis and treatment services. Only through a collaborative and inclusive effort at the clinical, social, and political levels can we hope to significantly improve the overall well-being of the Spanish population and mitigate the devastating impact of eating disorders.

## Figures and Tables

**Table 1:** Sociodemographic characteristics of sample (*n* = 9913).

	Mean ± SD or *n* (%)
Male	1650 (16.64%)
Female	8263 (83.36%)
Age (years)	39.35 ± 5.57
Male Age (years)	39.87 ± 5.55
Female Age (years)	39.25 ± 5.58
Level of education	
Basic education	2703 (27.27%)
Higher education	7210 (72.73%)
Income level	
Low	4032 (40.67%)
Medium–high	5430 (54.78%)
Do not know/no answer	451 (4.55%)
Municipality	
<2000	444 (4.48%)
2000–10,000	1603 (16.17%)
>10,000	7866 (79.35%)

Note: SD = Standard Deviation.

**Table 2:** Distribution of the different eating disorders in the study population.

Diagnosed Eating Disorder	
No ED	9582 (96.66%)
Anorexia nervosa	36 (0.36%)
Bulimia nervosa	53 (0.53%)
More than one ED	48 (0.48%)
Other ED	194 (1.97%)

Note: ED = diagnosed eating disorder; Other ED = diagnosed binge-eating disorders or eating disorders declared but not specified in the survey by the participant.

**Table 3:** Symptoms of possible eating disorders (*n* = 9913).

	Never	Rarely	Occasionally	Frequently	Very Often	Always
Obesophobia	845 (8.50%)	1552 (15.66%)	2605 (26.28%)	2409 (24.30%)	1421 (14.33%)	1081 (10.90%)
No control	1393 (14.05%)	2851 (28.76%)	2880 (29.05%)	1553 (15.67%)	914 (9.22%)	322 (3.25%)
Body image	287 (2.90%)	1614 (16.28%)	3011 (30.37%)	2691 (27.15%)	1378 (13.90%)	932 (9.40%)

**Table 4:** Symptoms of eating disorders in individuals with diagnosed anorexia or bulimia (*n* = 89).

	Never	Rarely	Occasionally	Frequently	Very Often	Always
Obesophobia	3	4	10	21	26	25
	(3.37%)	(4.49%)	(11.24%)	(23.60%)	(29.21%)	(28.09%)
No control	7	13	17	23	19	10
	(7.87%)	(14.61%)	(19.10%)	(25.84%)	(21.35%)	(11.24%)
Body image	1	4	13	29	22	20
	(1.12%)	(4.49%)	(14.61%)	(32.58%)	(24.72%)	(22.47%)

**Table 5:** Dietary and lifestyle habits in healthy population and people with anorexia or bulimia.

Numerical Variable	Healthy Population	Anorexia	Bulimia	*p*-Value ^$^
BMI	24.34 ± 4.39	20.45 ± 2.27	25.25 ± 5.57	H-A (<0.001)
				H-B (1.00)
				A-B (<0.001)
IASE	54.18 ± 9.95	52.25 ± 10.13	51.57 ± 12.03	0.21 ^‡^
Fried food	2.15 ± 0.78	1.86 ± 0.76	2.04 ± 0.78	H-A (0.06)
				H-B (0.87)
				A-B (0.81)
Fast food	2.33 ± 0.75	1.86 ± 0.83	2.42 ± 0.72	H-A (<0.001)
				H-B (1.00)
				A-B (<0.001)
Ultra-processed food	2.46 ± 0.93	2.12 ± 1.04	2.44 ± 0.95	0.40 ^‡^
Fish	1.86 ± 0.49	1.85 ± 0.55	1.83 ± 0.49	0.91 ^‡^
Water	3.40 ± 0.63	3.50 ± 0.61	3.49 ± 0.61	0.37 ^‡^
Sugary soft drinks	1.37 ± 0.65	1.33 ± 0.68	1.60 ± 0.84	0.07 ^‡^
Juice	1.20 ± 0.50	1.28 ± 0.70	1.17 ± 0.43	0.94 ^‡^
Coffee and energy drinks	1.79 ± 0.72	1.94 ± 0.86	1.81 ± 0.59	0.54 ^‡^
Sedentary lifestyle	1.66 ± 0.87	1.46 ± 0.76	1.55 ± 0.86	0.95 ^‡^
Self-perceived health	3.82 ± 0.83	3.72 ± 0.88	3.58 ± 0.89	0.06 ^‡^
Sport	145.71 ± 165.86	267.71 ± 203.37	160.19 ± 160.14	H-A (<0.001)
				H-B (0.77)
				A-B (0.047)
Sleeping hours	2.44 ± 0.73	2.31 ± 0.75	2.08 ± 0.70	H-A (1.00)
				H-B (<0.001)
				A-B (0.33)
Getting up rested	2.52 ± 0.59	2.36 ± 0.64	2.34 ± 0.59	H-A (0.34)
				H-B (0.13)
				A-B (1.00)
Sleep quality	3.31 ± 1.03	2.94 ± 1.04	2.89 ± 1.44	H-A (0.05)
				H-B (0.14)
				A-B (1.00)
Smoking	1.22 ± 0.62	1.25 ± 0.77	1.28 ± 0.66	0.46 ^‡^
Alcohol	1.74 ± 0.88	1.64 ± 0.87	1.72 ± 1.03	0.54 ^‡^
Getting drunk	1.04 ± 0.26	1.06 ± 0.23	1.23 ± 0.64	H-A (1.00)
				H-B (<0.001)
				A-B (0.14)
Night outings	1.09 ± 0.31	1.06 ± 0.23	1.11 ± 0.38	0.78 ^‡^

Note: ^$^ Kruskal–Wallis test with Bonferroni correction (pairwise comparisons). ^‡^ Not significant differences between groups.

**Table 6:** Prevalence according to different sociodemographic groups.

Kerrypnx	Healthy Population *n* (%)	Anorexia *n* (%)	Bulimia *n* (%)	*p*-Value ^&^
Men	1641 (17.13%)	1 (2.78%)	2 (3.77%)	<0.001
Women	7941 (82.87%)	35 (97.22%)	51 (96.23%)	
	3.51 × 10^−3 §^	0.14 ^§^	0.06 ^§^	
High education	6985 (72.9%)	30 (83.33%)	40 (75.47%)	0.19
Low education	2597 (27.1%)	6 (16.67%)	13 (24.53%)	
Low income	5273 (57.60%)	16 (50.00%)	26 (52.00%)	0.54
Medium–High income	3882 (42.40%)	16 (50.00%)	24 (48.00%)	
Small city	423 (4.41%)	2 (5.56%)	3 (5.66%)	0.55
Medium city	1529 (15.96%)	9 (25.00%)	10 (18.87%)	
Big city	7630 (79.63%)	25 (69.44%)	40 (75.47%)	

Note: *n* and percentages per column; ^&^ = χ^2^ test; ^§^ = Adjusted *p*-value with Bonferroni correction.

## Data Availability

The data presented in this study are available upon reasonable request to the corresponding author. Data is not publicly available due to the data are part of an ongoing study.
